# Exploring artificial intelligence techniques to research low energy nuclear reactions

**DOI:** 10.3389/frai.2024.1401782

**Published:** 2024-08-23

**Authors:** Anasse Bari, Tanya Pushkin Garg, Yvonne Wu, Sneha Singh, David Nagel

**Affiliations:** ^1^Courant Institute of Mathematical Sciences, New York University, New York, NY, United States; ^2^School of Engineering and Applied Science, George Washington University, Washington, DC, United States

**Keywords:** low energy nuclear reactions (LENR), nuclear energy, predictive analytics, artificial intelligence, unsupervised learning

## Abstract

The world urgently needs new sources of clean energy due to a growing global population, rising energy use, and the effects of climate change. Nuclear energy is one of the most promising solutions for meeting the world’s energy needs now and in the future. One type of nuclear energy, Low Energy Nuclear Reactions (LENR), has gained interest as a potential clean energy source. Recent AI advancements create new ways to help research LENR and to comprehensively analyze the relationships between experimental parameters, materials, and outcomes across diverse LENR research endeavors worldwide. This study explores and investigates the effectiveness of modern AI capabilities leveraging embedding models and topic modeling techniques, including Latent Dirichlet Allocation (LDA), BERTopic, and Top2Vec, in elucidating the underlying structure and prevalent themes within a large LENR research corpus. These methodologies offer unique perspectives on understanding relationships and trends within the LENR research landscape, thereby facilitating advancements in this crucial energy research area. Furthermore, the study presents LENRsim, an experimental machine learning tool to identify similar LENR studies, along with a user-friendly web interface for widespread adoption and utilization. The findings contribute to the understanding and progression of LENR research through data-driven analysis and tool development, enabling more informed decision-making and strategic planning for future research in this field. The insights derived from this study, along with the experimental tools we developed and deployed, hold the potential to significantly aid researchers in advancing their studies of LENR.

## 1 Introduction

Low Energy Nuclear Reactions is a promising field of research and development for producing clean and sustainable energy. LENR harnesses nuclear reactions at ambient temperatures to generate energy with remarkable potential for commercialization. The potential benefits of LENR technology, include the prospect of obtaining cost-efficient energy production, minimal radiation emission during operation, a lack of significant radioactive waste generation, and negligible greenhouse gas emissions. Due to these multiple attractive features, LENR offers significant promise for future energy technologies (Nagel, 2012; Ruer, 2020).

While interest in LENR and its potential impacts continues to grow, the literature lacks systematic methods for comprehensively extracting and analyzing patterns across the various, intricate aspects of this field, which impedes progress toward a holistic understanding. As researchers explore the vast and diverse body of LENR publications, the need to efficiently consolidate and comprehend this information grows more pressing. This paper addresses this critical gap by introducing a new machine learning framework employing topic modeling techniques to extract latent themes from LENR literature. We sought to develop a document similarity tool to facilitate efficient navigation and comparison of relevant research findings. By developing and releasing such tools, our work provides a valuable resource for researchers and practitioners seeking to explore, synthesize, and build upon the collective knowledge within this expanding field.

The AI tools we sought to develop focus on identifying commonalities and similar semantics among LENR research papers, and uncovering patterns and trends that might not be realized using traditional manual approaches. By harnessing the power of machine learning and natural language processing, we intend to reveal novel connections and correlations that can shape our understanding of LENR and its potential in the clean energy landscape. Through these data-driven techniques, we aim to provide a deeper understanding of its underlying principles and potential applications. Hence, the results of our work will facilitate and speed both research and commercialization of LENR.

Our study accomplishes this by using existing and new AI tools for the development and applications in the field, with a particular emphasis on two crucial aspects:

- We identify prevalent themes through topic modeling of LENR literature. This helps researchers to know emerging trends and recurring themes within the LENR literature, which allows a more in-depth understanding of the current state of LENR research.
- Moreover, we introduce a machine learning tool designed to identify semantically similar research studies in the domain of LENR. This tool enables efficient retrieval and navigation of relevant literature within the vast corpus of LENR research. Researchers can easily identify related studies, compare findings, and track the evolution of ideas over time, thereby accelerating the pace of discovery and innovation.

To help energy scientists study and harness LENR’s potential, we designed an experimental machine learning tool that integrates vector embeddings, topic modeling, and two-phase retrieval that aims at help providing valuable insights from the vast nuclear energy research literature. By providing a user-friendly tool for exploration and inquiry, we aspire to drive advancements in LENR technology, ultimately contributing to a sustainable and greener future for our planet.

In the subsequent sections of this paper, we first dive into a structured approach to our research methodology. Section 2 presents the background work, Section 3 outlines the methods employed for data collection, while Section 4 discusses the preprocessing techniques utilized to ensure data quality. Section 5 elucidates our implementation of topic modeling, encompassing a thorough examination of various algorithms, clustering techniques, and comprehensive evaluation of results. Following this, Section 6 elaborates on the development of a document similarity tool, which leverages the insights derived from the clustering techniques elucidated in Section 4.

## 2 Background and related work

Topic modeling of research publication data categorizes and uncovers emerging trends in scientific articles and reports, promoting efficient knowledge exploration and research progress. In recent years, studies across various fields have employed this technique to get a better understanding of their respective research areas. Numerous studies have embraced topic modeling to acquire deeper insights into diverse domains. For instance, Fan et al. (2023) employed topic modeling to explore the potential of large language models (LLMs) research and its practical applications. Likewise, Egger and Yu (2022) compared various topic modeling techniques on X (formerly known as Twitter) data to facilitate social science research and extract valuable insights into human interactions during the COVID-19 pandemic. A similar study applying text-based machine learning algorithms to the LENR corpus has not been done yet. It could provide valuable insights into this niche area and contribute significantly to advancing research in the field.

Prior to the emergence of neural network-based models, transforming text into low-dimensional embeddings relied on techniques like decomposing co-occurrence matrices or probabilistic models such as Linear Dirichlet Allocation (LDA). Recent advancements in the field of Natural Language Processing (NLP) have introduced novel approaches to topic modeling, such as BERTopic (Grootendorst, 2022) and Top2Vec (Angelov, 2020). These methods provide systematic pipelines for topic modeling, incorporating methods like embedding, clustering, and topic extraction, offering flexibility in choosing specific techniques and models for each module.

Embedding is a way to represent paragraphs as numerical vectors such that similar meanings are closer in the vector space. The selection of embeddings plays a crucial role in determining the accuracy and effectiveness of a topic model, as embeddings capture semantic and contextual information, thereby improving the understanding of word and document relationships. Doc2Vec (Le and Mikolov, 2014) is one such document embedding model, extending the widely used Word2Vec model. It employs shallow neural networks to represent documents as unique vectors in the embedding space. On the other hand, Sentence BERT, based on transformer architecture, leverages deep learning techniques to generate sentence-level embeddings, gaining popularity in recent times (Reimers and Gurevych, 2019). This paper builds upon these algorithms to derive hidden insights from the field of Low Energy Nuclear Reactions.

## 3 Dataset

This study focuses exclusively on a type of nuclear energy known as Low Energy Nuclear Reactions (LENR) research and relies on a dataset specific to LENR. We primarily utilized the comprehensive LENR-CANR publicly available bibliography hosted by Rothwell (2002)^1^ as our main data source, which includes over 4,743 entries. Each entry includes metadata such as title, author list, publication year, source of publication, abstract, and PDF links, with 2,174 directly linked documents hosted by LENR-CANR.

This compilation covers a unique and diverse range of publication types from the late 1980s to recent years, including journal articles, conference presentations, white papers, books, and newsletters, across various disciplines. Of the 4,743 entries, the 2,174 directly linked PDFs were programmatically collected from the LENR-CANR server for analysis. Furthermore, we expanded the dataset by manually retrieving 1,250 publicly available papers and reports from sources such as Google Scholar, ScienceDirect, Nature, IEEE Xplore, and preprint repositories, aggregating a comprehensive unique database of 3,424 LENR documents. This expanded dataset, spanning over 200,000 pages, significantly enhances our capacity to investigate publication trends, employ text analytics for topic modeling, and explore intricate conceptual relationships within the scattered LENR literature. This augmented dataset not only aims to help deepen our understanding of the field’s development but also aims to provide a robust foundation and LENR data hub for advanced text analytics, enabling comprehensive insights through in-depth textual analysis and exploration.

For the purpose of this study, we used titles and abstracts extracted from the LENR literature. Out of a total of 3,424 LENR-related documents, 3,034 of these documents underwent additional preprocessing procedures to facilitate analytical processes, which are described in the next section. The remaining 400 documents were excluded due to the absence of clear abstracts, such as those typically found in PowerPoint presentations.

## 4 Data preparation

The research papers and articles were first converted into a text format for further analysis. For this study, we only used the title and abstract for each document in the dataset. Titles and abstracts are typically concise summaries that provide an overview of the research conducted in the document. By focusing on these sections, we aim to extract the core information and meaning of each document, enabling us to effectively analyze and compare the content across the entire corpus. To ensure data cleanliness and improve the quality of subsequent processing, the texts underwent a cleaning phase. This involves the removal of stop words and punctuations, which do not contribute to the semantic and linguistic meaning of the text. Additionally, the list of stop words included commonly occurring search terms such as “LENR” and “cold fusion” to eliminate their influence on the analysis. Furthermore, a lemmatization process was applied to the text to enhance the analysis by capturing the underlying concepts and relationships between words more effectively. Lemmatization reduces words to their base form, allowing for a comprehensive exploration of the text and facilitating better insights for subsequent research (Bird et al., 2009).

## 5 Topic modeling

**Figure 1:**
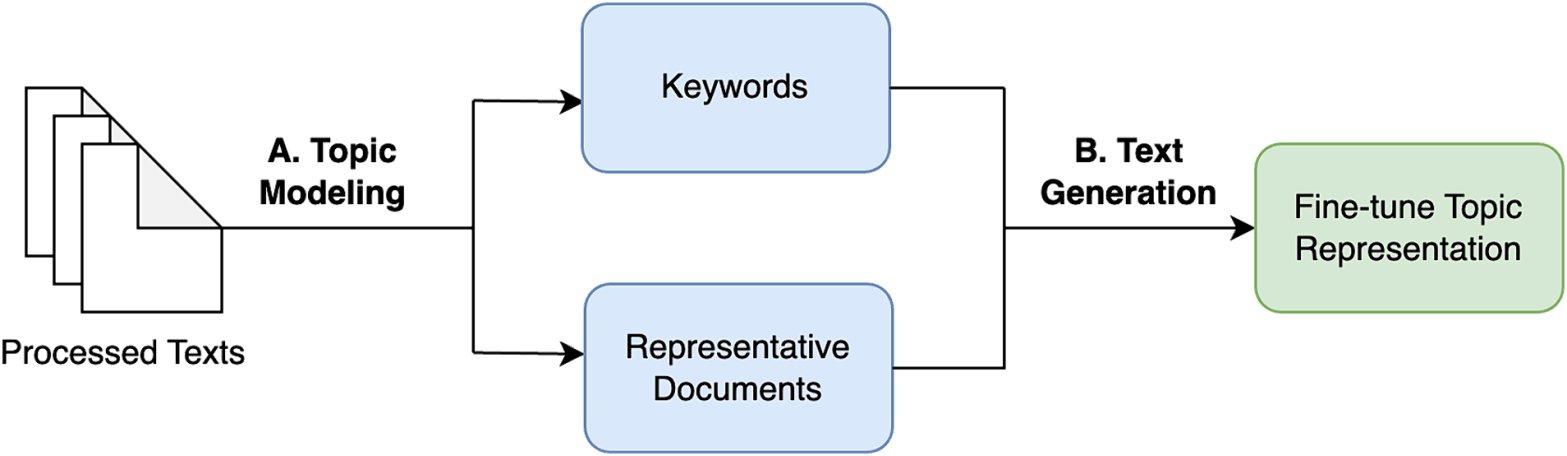
Systematic approach to topic modeling.

Topic modeling is the extraction of topics or themes from large text data to uncover hidden semantic structures, and cluster similar groups of documents together in an unsupervised manner, as shown in Figure 1. This involves generating a set of keywords that effectively capture the essence of each topic using machine learning techniques. These keywords serve as indicators and help identify the main themes and subject areas associated with each topic. The model also provides representative documents that offer the most comprehensive and insightful descriptions of each topic. These documents are chosen based on their relevance and ability to encapsulate the key aspects of the respective topics.

In Section 5.1, we provide an in-depth exploration of the topic modeling algorithms employed in our study. This is followed by a discussion on the refinement of topic labels through the utilization of text generation models in Section 5.2. Furthermore, Section 5.3 entails the evaluation of the topic models and associated clustering algorithms, offering insights into their effectiveness and performance within the context of our research.

### 5.1 Topic modeling algorithms

To achieve our research objectives, we conducted an in-depth analysis of the LENR corpus using the aforementioned topic models. LDA, Top2Vec, and BERTopic were each applied to the dataset, enabling the extraction and identification of latent topics from the collection of papers. The subsequent paragraphs delve into the intricacies of each model, elucidating their methodologies and applications. Section 5.3.2 presents a comparative evaluation of these models, delineating their respective strengths and limitations in discerning topical structures within the LENR literature.

#### 5.1.1 Latent Dirichlet Allocation (LDA)

Latent Dirichlet Allocation (LDA) is one of the most commonly used topic modeling algorithms. The LDA is a hierarchical Bayesian model with three levels, representing each item in a collection as a finite mixture over a set of underlying topics. These topics are, in turn, modeled as an infinite mixture over a set of topic probabilities. In the domain of text modeling, these topic probabilities serve as an explicit representation of a document’s content and themes (Blei et al., 2003; Maier et al., 2018). Despite its popularity, LDA exhibits certain weaknesses. One such drawback is the necessity to fine-tune the number of topics as a model parameter. Additionally, the method relies on a bag-of-words representation of documents, which disregards the semantics and the sequential ordering of words (Angelov, 2020).

In this research study, Latent Dirichlet Allocation (LDA) was utilized as a benchmark to conduct a comparative analysis with advanced topic modeling methodologies. The application of LDA in this investigation involved a preprocessing step, wherein tokenized text data was transformed into a bag-of-words representation. This representation portrays each document as an unordered collection of words along with their respective frequencies. The LDA modeling was facilitated through the utilization of gensim’s Python API (Řehůřek and Sojka, 2010).

Topic sets were constructed with varying numbers of topics, denoted as *K* = {5, 10, 15, 20, 30, 50}, while maintaining the default value for the number of top keywords parameter, set at 10. The coherence of the resulting topics was assessed using Pointwise Mutual Information (UMass) (Mimno et al., 2011), C_NPMI_ (Aletras and Stevenson, 2013), and C_V_ (Röder et al., 2015) measures, alongside their relevance, aiming to gauge the efficacy of LDA in capturing latent structures within the dataset. Subsequent analysis revealed that a *K* value of 10 yielded the most favorable outcomes.

The use of LDA in this comparative framework serves as a baseline, allowing for a better understanding of its performance in contrast to more contemporary approaches to topic modeling.

#### 5.1.2 Top2Vec

Top2Vec distinguishes itself from conventional methodologies, such as LDA, due to its distinctive employment of a neural network-based model for automatic topic discovery and representation within a given corpus of documents. This innovative approach enables Top2Vec to effectively capture semantic meaning and contextual relationships among words, effectively overcoming the limitations of traditional bag-of-words techniques. Leveraging this advantage, Top2Vec transforms both documents and word embeddings into a unified vector space, facilitating an efficient and comprehensive exploration of topic clusters and their evolution over time (Angelov, 2020).

In this study, the generation of word and document embeddings utilized a pre-trained embedding model, Doc2Vec. To address the inherent sparsity of the vector space, primarily comprising zero values, a dimension reduction step was performed prior to density clustering. Uniform Manifold Approximation and Projection (UMAP) was employed to significantly reduce dimensions, followed by Hierarchical Density-Based Spatial Clustering of Applications with Noise (HDBSCAN) to identify dense regions within the documents. Subsequently, the topic vectors were derived by computing the centroids of the document vectors in the original dimension, corresponding to each dense area found through the clustering process (Egger and Yu, 2022).

We employed the Top2Vec python package with default parameters to implement Top2Vec. Our experimentation involved exploring different types of embeddings, including document embeddings and various sentence embeddings. Our findings revealed that doc2vec yielded superior results in terms of coherence measures compared to other embedding methods.

#### 5.1.3 BERTopic

**Figure 2:**
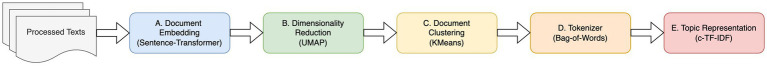
BERTopic algorithm flow **(A)** numerically represent documents using embeddings, **(B)** reduce dimensionality, **(C)** cluster reduced embeddings, **(D)** tokenize topics, **(E)** extract topic words.

BERTopic is similar to Top2Vec in terms of algorithmic structure. Figure 2 shows various steps in the algorithm pipeline. BERTopic employs the Sentence-BERT (SBERT) framework for the embedding step, enabling the conversion of sentences and paragraphs into dense vector representations using pre-trained language models. In our study, we evaluated the accuracy of three distinct pre-trained embeddings: Word2Vec, “all-MiniLM-L6-v2,” (Reimers and Gurevych, 2019) and “e5-base-v2” (Wang et al., 2023). For data clustering, various clustering algorithms were experimented like HDBSCAN, KMeans and Agglomerative algorithms. While HDBSCAN, a density-based approach, yielded numerous outliers for our dataset, our clustering task necessitated clear-cut categorization. Therefore, KMeans emerged as the more fitting algorithm. Additionally, BERTopic incorporates the class-based term frequency-inverse document frequency (c-TF-IDF) algorithm, which assesses the significance of terms within a cluster and creates corresponding term representations (Grootendorst, 2022). The higher the c-TF-IDF value for a term, the more indicative it is of its associated topic.

We employed the BERTopic Python package to conduct experiments with various embedding models and clustering algorithms. For dimensionality reduction, we utilized the UMAP technique, known for its ability to effectively capture both local and global structures in high-dimensional embedding data when projecting it into a lower-dimensional space. We used the bag-of-words approach for the tokenizer phase, followed by the topic representation phase, where we utilized the default parameters for the class-based TF-IDF technique. Section 5.3 presents the evaluation metrics and comparisons of different clustering and embedding techniques.

### 5.2 Fine-tune topic representation

**Figure 3:**
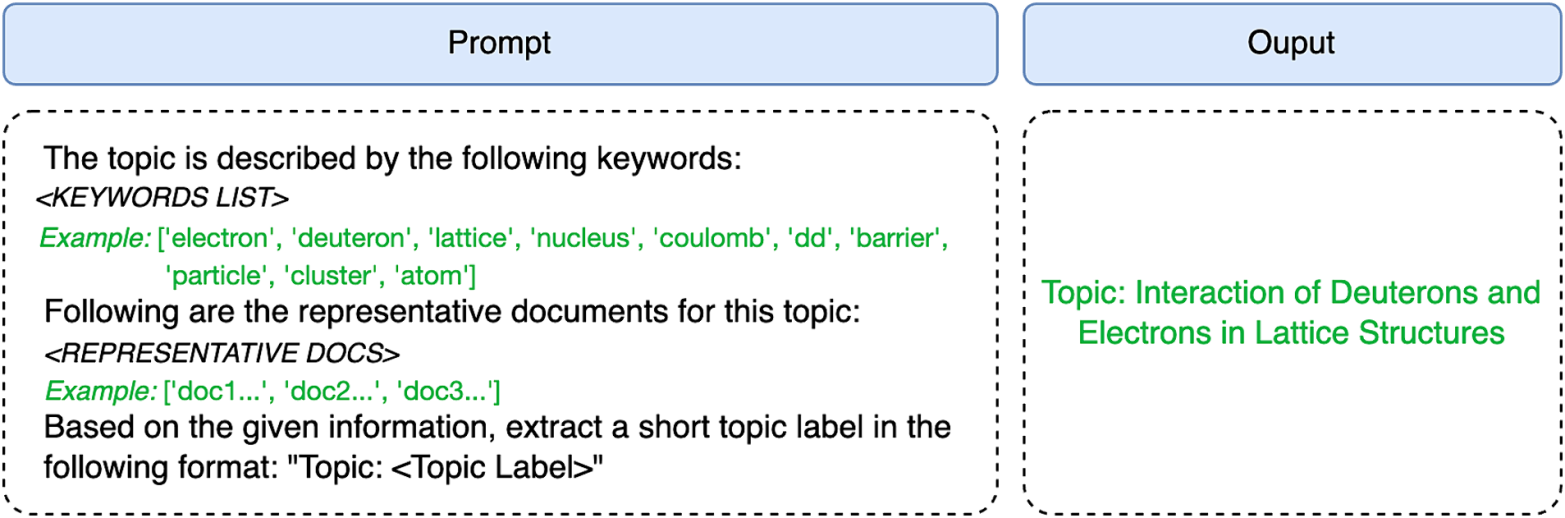
An example of fine-tuning topic labels. Left: A standard prompt with keywords and representative documents input for a specific topic. Right: Fine-tuned topic label for human interpretability.

Topic Modeling offers keywords associated with each document cluster, as well as representative cluster documents. While these keywords are significant in describing particular groups of topics, they may lack human interpretability. To enhance topic representation, we employed a text generation model, specifically leveraging large language models such as GPT-3.5. By inputting the set of keywords and representative documents into the text generation model, we prompted the model to produce text that seamlessly aligns with each specific topic. Through this approach, our research aimed to achieve accurate and cohesive representations of the topics, resulting in informative and contextually appropriate textual output. For this purpose, we utilized OpenAI’s chat completion API, employing the ‘gpt-3.5-turbo’ model with default parameters, and prompt as depicted in Figure 3.

### 5.3 Evaluation

In this section, we elaborate on the evaluation criteria and present the results obtained from the application of different clustering algorithms and topic models employed in our study.

#### 5.3.1 Data clustering algorithms

We assess the performance of varying clustering algorithms employed for grouping similar documents together to extract topics, as illustrated in Figure 2, Part C. In the context of assessing document clustering techniques, evaluation metrics are commonly categorized into two distinct groups: intrinsic and extrinsic measures. Intrinsic metrics, which encompass parameters like cluster separation and cohesion, possess the distinctive attribute of not relying on a predefined reference or ground truth label. These metrics primarily aim to capture the intrinsic properties of clusters by quantifying the interplay of data points within and across clusters, shedding light on the inherent structure of the data (Curiskis et al., 2020). To evaluate the various clustering algorithms three measures were used: Silhouette Score, Davies-Bouldin Index, and Calinski-Harabasz Index.

- Silhouette Score: This measures the cluster separation and cohesion. It is computed as the difference between the average distance from a data point to its own cluster (cohesion) and the average distance to the nearest neighboring cluster (separation), normalized by the maximum of these two values, resulting in a score between −1 and + 1, where +1 represents well-separated clusters, and − 1 represents poor clustering (Rousseeuw, 1987; Pedregosa et al., 2011).
- Davies-Bouldin Index: This measures the average similarity between clusters. It is computed by calculating the average similarity ratio for each cluster with the cluster that is most similar to it. For each cluster, it finds the ratio of the average distance between data points within the cluster (intra-cluster similarity) to the distance between the centroids of the clusters (inter-cluster similarity). The Davies-Bouldin Index is then the maximum of these similarity ratios across all clusters. The lower the value, the better the cluster quality (Davies and Bouldin, 1979; Pedregosa et al., 2011).
- Calinski-Harabasz Index: Also known as Variance Ratio Criterion, it measures the ratio of between-cluster variance to within-cluster variance. Higher values suggest better separation between clusters (Calínski and Harabasz, 1974; Pedregosa et al., 2011).

Data clustering algorithms, like KMeans, necessitate the specification of the number of clusters as a parameter. To determine the ideal cluster count, we employed the elbow method. This involved calculating the within-cluster sum of squares (WCSS) across varying cluster counts. The point at which the WCSS demonstrates a diminished rate of decline designates the optimal cluster count, achieving an equilibrium between mitigating intra-cluster distances and avoiding excessive cluster count, prone to overfitting. For our investigation, the elbow method was applied across a spectrum of 2–25 clusters. Following careful analysis, we selected six as the optimal number of clusters.

Table 1 provides a performance evaluation of clustering algorithms for document clustering: Silhouette Score (SC), Davies-Bouldin Index (DBI) and Calinski-Harabasz Index (CHI). The agglomerative clustering algorithm demonstrates poor performance across all the given metrics, indicating suboptimal cluster separation and cohesion. This performance disparity may be attributed to the hierarchical nature of agglomerative clustering, leading to nested and overlapping clusters, and its sensitivity to noise and outliers in high-dimensional feature spaces. The KMeans and DBSCAN algorithms outperformed the other methods on all three evaluation measures. Notably, these algorithms yielded comparable evaluations with regard to both the Silhouette Score and the Davies-Bouldin Index, indicating that they produce clusters characterized by equivalent levels of cluster cohesion and cluster similarity. However, the KMeans algorithm outperforms DBSCAN with respect to the cluster separation metric (Calinski-Harabasz Index). Furthermore, when utilizing DBSCAN with a range of hyperparameters, a substantial proportion of documents were assigned to an outlier cluster. This phenomenon may be attributed to the high-dimensional and sparse nature of the feature representations, which can lead to suboptimal results when employing density-based clustering algorithms (Curiskis et al., 2020).

**Table 1:** Clustering models evaluation.

Model	Silhouette Score	Davies-Bouldin Index	Calinski-Harabasz Index
KMeans	0.032	3.91	71.32
DBSCAN	0.034	3.86	31.99
HDBSCAN	0.018	3.26	22.15
Agglomerative clustering	0.017	4.77	52.29

#### 5.3.2 Topic modeling algorithms

The evaluation of topic models in this study involves two key measures: Topic Coherence and Topic Diversity. Topic Coherence assesses the quality of the topic-word distribution (Qiang et al., 2020). To compute topic coherence, we utilized the Normalized Pointwise Mutual Information (NPMI) method (Bouma, 2009). Essentially, a coherent topic is expected to have highly associated words, resulting in an NPMI score ranging between −1 and 1, where 1 signifies a perfect association. On the other hand, Topic Diversity quantifies the percentage of unique words among the top 25 words across all topics. If the diversity value is close to 0, it indicates redundant topics, while a value closer to 1 suggests a broader range of topics (Dieng et al., 2020).

Table 2 elaborates the Topic Coherence (TC) and Topic Diversity (TD) scores for various topic models and embedding techniques, presented as an average over three runs. Among the evaluated methods, LDA excels in topic coherence, while Top2Vec performs exceptionally well in terms of topic diversity. However, when employing BERTopic with Microsoft’s e5-base-v2 embeddings, superior scores are achieved for both measures. This outperformance of LDA and Top2Vec is observed on the preprocessed title and abstract LENR dataset. It is clear that the BERTopic model with e5-base-v2 embeddings, and KMeans clustering delivered the best performance on the LENR dataset.

**Table 2:** Topic models evaluation.

Model	Topic coherence	Topic diversity
LDA	0.06	0.66
Top2Vec: Doc2Vec	−0.015	0.99
BERTopic: Word2Vec	−0.01	0.92
BERTopic: all-MiniLM-L6-v2	−0.05	0.98
BERTopic: e5-base-v2	0.14	0.98

**Figure 4:**
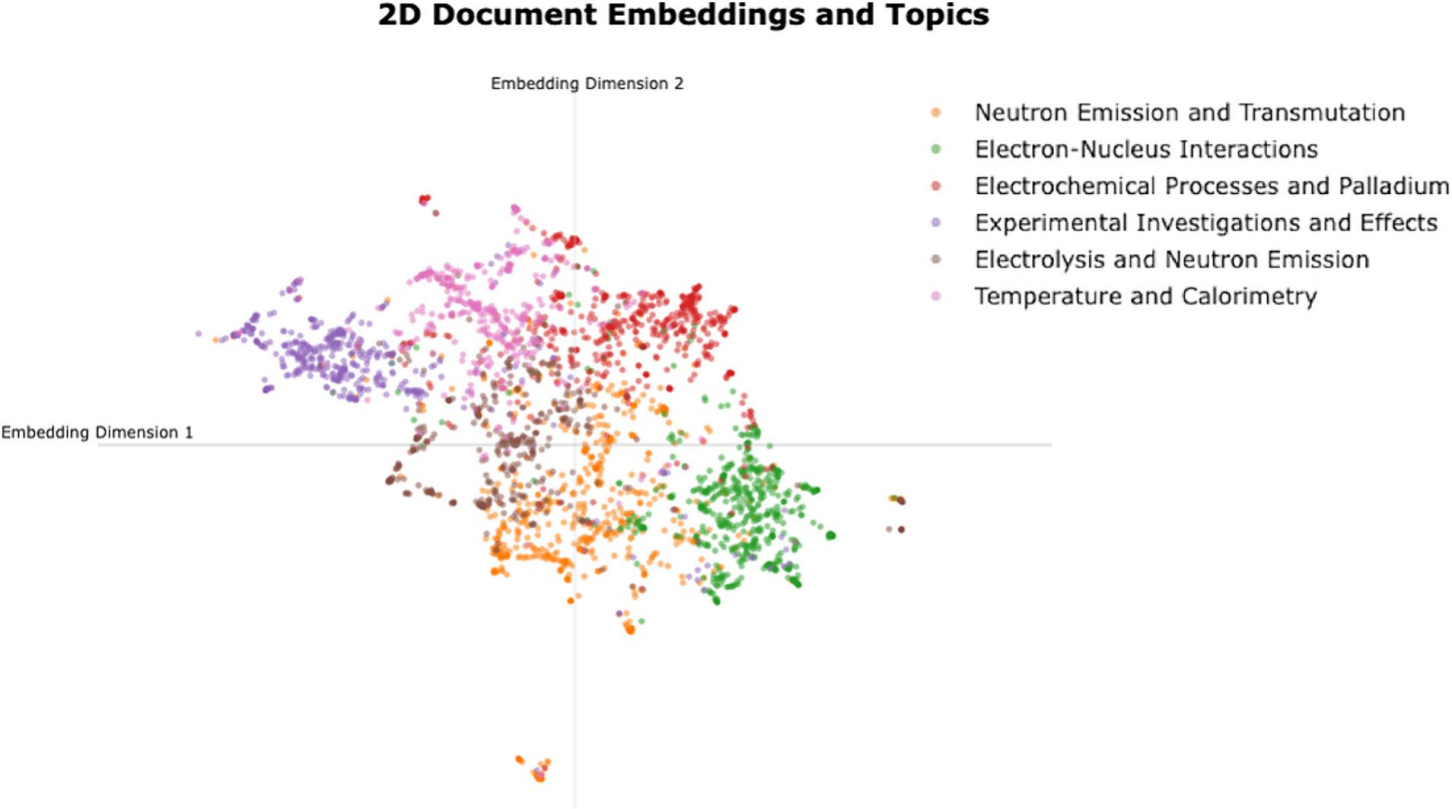
2D Map of LENR research publications embeddings with research topics.

Figure 4 shows the 2D representation of LENR document embeddings, clustered within each listed topic. We obtained sentence embeddings of each document using a pre-trained BERT model (Microsoft’s “e5-base-v2” embeddings). This enables the capture of semantic relationships and contextual information in a 768-dimensional space. To facilitate the visualization of the clusters, the embeddings are reduced to 2-dimensional space using UMAP. Each graph point represents a unique publication, color-coded to represent cluster topics. The X and Y axes represent sentence embeddings in the 2-dimensional space. The accompanying legend highlights thematic associations that are extracted using the BERTopic topic modeling approach as mentioned in Section 5.1.3. The topics listed in the legend are the same as those in Table 3, with their names shortened for visibility purposes.

The top 10 keywords and documents that best represent the topic clusters were used to derive fine-tuned topic representations to generate more human-interpretable research themes using the ChatGPT API. These representations are summarized in Table 3.

**Table 3:** Topics of LENR publications.

1. Neutron emission and deuterium transmutation
2. Interaction of deuterons and electrons in lattice structures
3. Hydrogen-palladium electrochemical system
4. Fleischmann experiment on low-energy reaction and excess heat
5. Electrolysis and tritium production in palladium cathode
6. Study of excess heat production in electrolytic cells using calorimetry and palladium cathode

## 6 Similar documents retrieval

This study provides an experimental machine learning-based tool to determine similar documents in the LENR research document corpus. Document similarity tools are instrumental in scientific research, serving a vital role in literature review, data clustering, data categorization, information retrieval, and data mining applications. Such tools streamline the literature review process by quickly identifying relevant papers. In clustering and categorization, these tools group similar documents, enabling effective organization and pattern recognition. For information retrieval, they aid in finding relevant documents based on queries, enhancing search engines and recommendation systems. Moreover, document similarity supports data mining tasks, uncovering insights and relationships within large datasets.

### 6.1 Algorithms

#### 6.1.1 Brute-force algorithm

The fundamental algorithm for calculating the K most similar documents to a given query document involves the computation of pairwise cosine similarity scores between the query document and every document within the provided corpus. Cosine similarity measures the cosine of the angle between two vectors in a high-dimensional space. In this context, each document is represented as a vector, also called word embeddings, and their cosine similarity scores reflect their alignment in this space. Given word embedding vectors A and B, the cosine similarity is calculated as:


$$\cos\left(\theta \right)=\frac{A\cdot B}{\left|A\right|\left|B\right|}$$

where θ is the angle between the vectors A and B. The values range from −1 to 1. The closer the angle between two embedding vectors, the larger the cosine value.

This is a naive algorithm that returns top K documents exhibiting the highest cosine similarity scores.

#### 6.1.2 Proposed two-phase algorithm

This algorithm leverages the BERTopic topic modeling algorithm discussed in Section 5.1.3 to determine the most semantically similar documents for a given query document.

During the pre-processing and clustering stages of the topic modeling process, the document embeddings and topic categories for each document are retained. BERTopic additionally supplies a compilation of representative documents for each document cluster. When a query document is provided by the user, it undergoes preprocessing procedures outlined in Section 4. Following the acquisition of embeddings for both the query document and the corpus, a two-phase similarity assessment is executed:

- The query document is matched against the representative documents from each topic cluster. The representative document exhibiting the best cosine similarity is designated as the cluster with the greatest document similarity.
- Subsequently, the query document is compared with each document within the selected cluster. The K documents yielding the best cosine similarity scores are chosen as the most pertinent and semantically similar data points.

### 6.2 Evaluation

The proposed algorithm is subjected to evaluation in comparison to the brute-force algorithm using two key metrics:

- The average cosine similarity score is computed for 10 distinct query documents. For each query document, the algorithm identifies the top five most similar documents. The resulting average values across all iterations are juxtaposed between the proposed and brute-force algorithms, and these findings are displayed in Table 4. It is evident from the results that the proposed algorithm exhibits a level of accuracy nearly equivalent to that of the brute-force algorithm.
- The performance of both algorithms is quantified in terms of the time required for each algorithm to identify the top similar documents. As illustrated in Table 4, the proposed algorithm demonstrates an average runtime of approximately one-fourth that of the brute-force algorithm. It is worth noting that this performance advantage is expected to become more pronounced as the size of the document corpus increases.

**Table 4:** Document similarity algorithm evaluation.

Algorithm	Cosine value	Performance (ms)
Brute-force algorithm	0.9	46.03
2-Phase algorithm	0.8925	11.86

**Figure 5:**
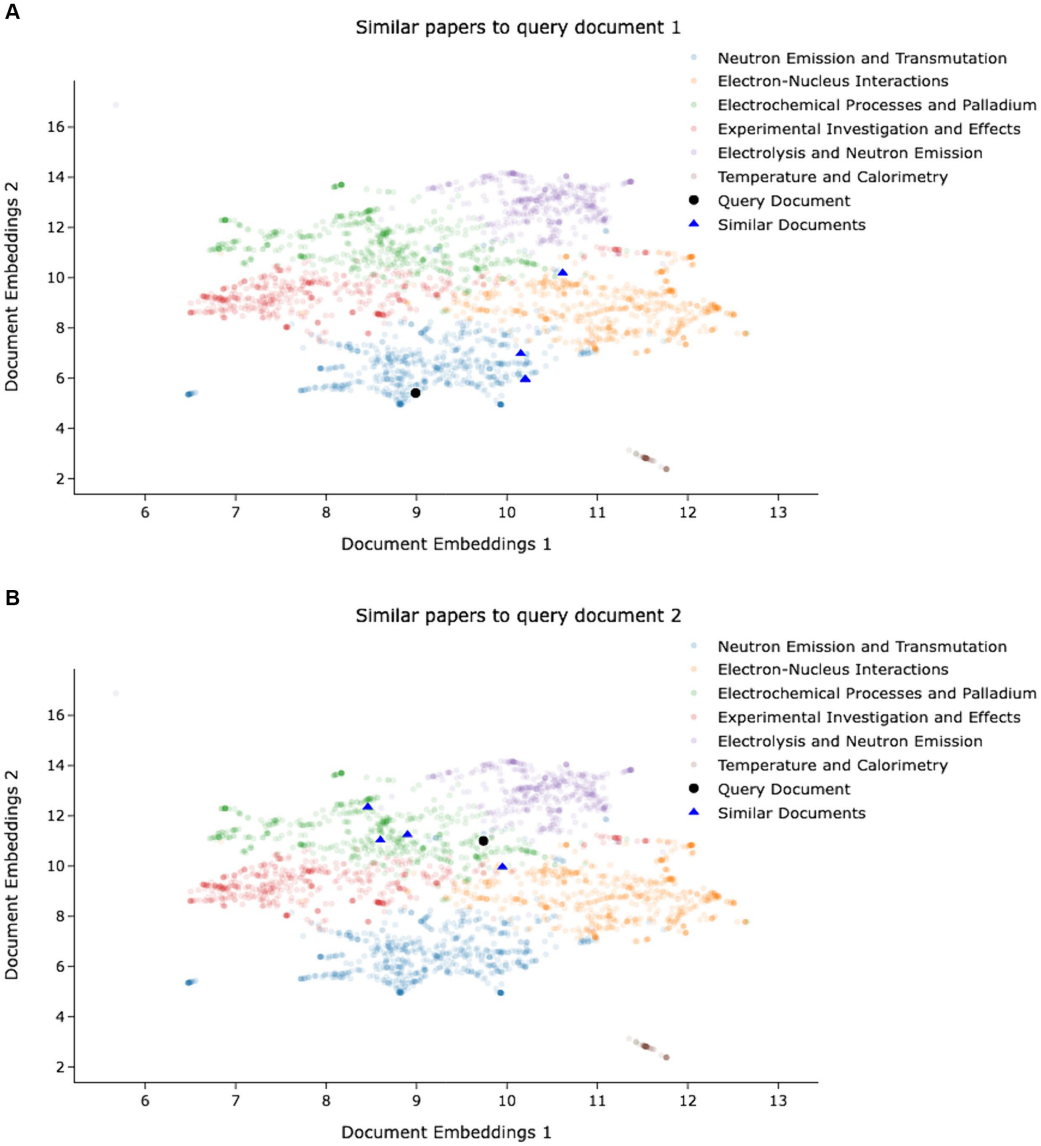
**(A)** Query 1 and similar document embeddings in 2D space. **(B)** Query 2 and similar document embeddings in 2D space.

We provide the following two examples to illustrate the results of the two-phase algorithm:

- The query document, titled “Binuclear Atoms: A Model to Explain Low Energy Nuclear Reactions,” offers a theoretical framework within the realm of low-energy nuclear reactions (LENR). This work introduces the novel concept of binuclear atoms, shedding light on the underlying mechanisms of this phenomenon and its potential applications in the fields of energy generation and nuclear physics. The proposed algorithm successfully identifies the five most similar documents as depicted in Figure 5A. Both the brute-force and proposed KMeans algorithms yield an average cosine similarity score of 0.882. Upon manual examination of the retrieved documents, it is conclusively established that they share a common thematic focus.
- Likewise, an additional query document, bearing the title “Can Water be the Origin of Excess Energy?” undertakes an exploration of the captivating hypothesis regarding water’s potential role as a source of energy, thoroughly investigating its implications in the domain of energy production and associated scientific phenomena. The proposed algorithm successfully identifies the five most similar documents, as represented in Figure 5B. The cosine similarity score for the naive Kmeans algorithm is 0.913, while the proposed algorithm achieves a comparable score of 0.91. Once again, through meticulous manual scrutiny of the retrieved documents, it is unequivocally ascertained that they converge upon a shared thematic focus.

While it might be expected to have similar articles to be close to each other in the embedding space, Figure 5 shows that this assumption may not be true. The observed lack of proximity among similar documents in the two-dimensional visualization space could be due to the inherent complexity and high dimensionality of the original embedding space. The reduction of dimensionality may lead to information loss and distortion of the underlying semantic relationships. Additionally, transformer-based embeddings, particularly sentence transformers designed to capture the entire semantics of a sentence or paragraph rather than focusing on individual tokens, encode nuanced semantic features that may not be effectively captured in a lower-dimensional projection. The intricate interplay of various sematic dimensions and the non-linear relationships between words and phrases could attribute to the scattered distribution of similar documents in the reduced space. The traditional visualization methods might not fully encapsulate the intricate structures present in the high-dimensional embeddings.

### 6.3 Dashboard

**Figure 6:**
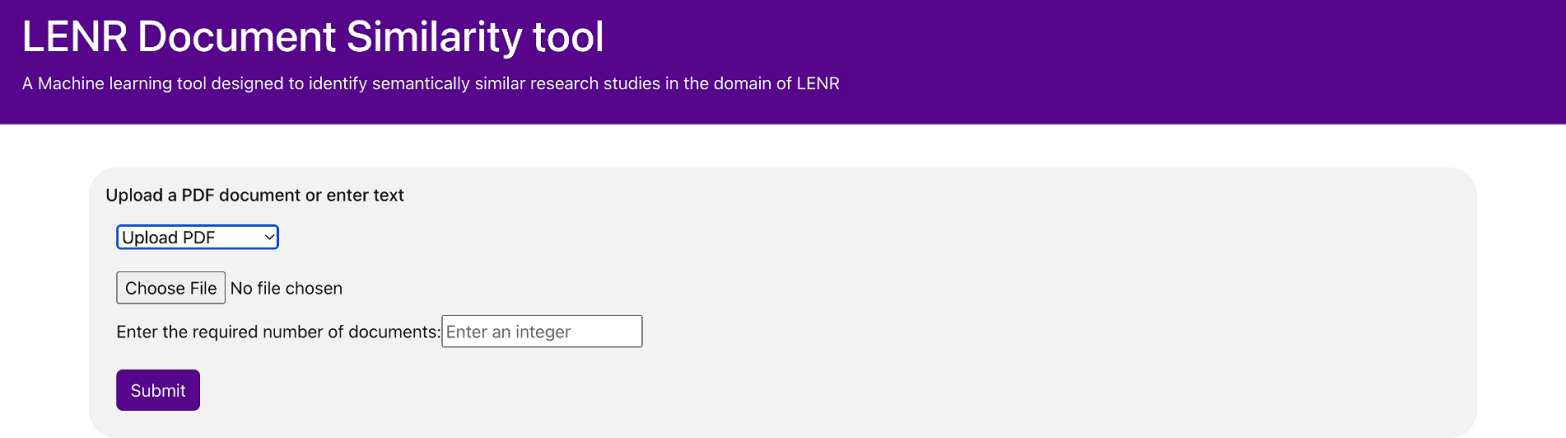
Interactive dashboard: enabling input of PDF Documents and the number of documents required.

**Figure 7:**
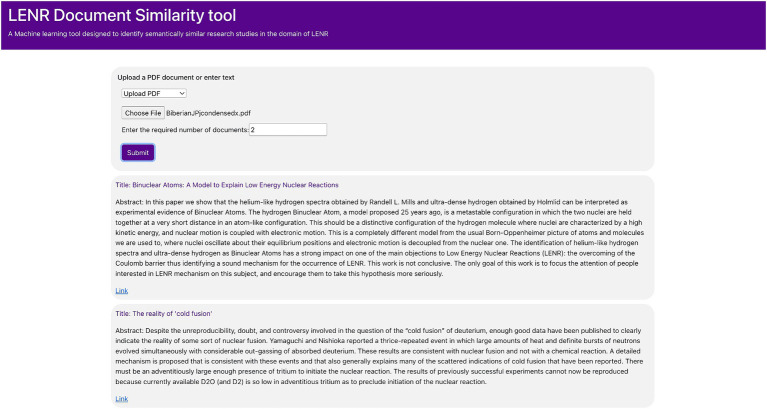
Highly similar documents: platform displaying matching papers with titles, abstracts, and document links.

The web application, LENRsim, has been developed employing React and Flask, integrating machine learning techniques to discern semantically similar research endeavors within the LENR domain. Illustrated in Figure 6, this platform offers users the flexibility to input their query document as either a PDF file or plain text, and number of similar documents to be retrieved (k). Upon submission of the document, the dashboard promptly provides the k research studies exhibiting the highest similarity to the query. Each displayed entry showcases the document’s title and abstract. Furthermore, users are given direct access to the original research paper through an accompanying link, as shown in Figure 7. The LENRsim tool is hosted at – lenrdashboard.com/document_similarity/similarity.html.

We have developed and deployed another interactive dashboard, which can be accessed at https://lenrdashboard.com/. This tool enables users to analyze document clusters and retrieve documents from each cluster (Supplementary Figure 1), provides distribution of LENR publications per year (Supplementary Figure 2), allows analysis of co-author information, among other features.

## 7 Conclusion

By seamlessly integrating AI, algorithms, and topic modeling, our study extracts invaluable insights from the vast pool of LENR research information. Data-driven approaches, especially topic models, provide fresh perspectives on research results and trends. Our user-friendly platform enables new means of exploration and inquiry, driving advancements in LENR science and technology.

This study contrasts the outcomes of three topic modeling algorithms utilizing distinct embedding techniques. Among the range of embedding-model combinations, BERTopic excels when coupled with pre-trained sentence embeddings, notably the recently introduced e5-based embeddings. This success is attributed to the innovative application of class-based TF-IDF for topic representation extraction. Additionally, text classification from topic modeling is leveraged to accelerate the retrieval of most similar documents for a given query. To provide users with this capability, a user-friendly web interface has been developed.

In this study, we utilized document titles and abstracts, considering the token limitations inherent in BERT-based models. However, we plan to extend our approach to encompass full texts in the next version. Although we did not test extensively on a large number of queries, our testing was sufficient to demonstrate that semantic retrieval is working effectively. Additionally, while we employed pre-trained sentence embeddings, fine-tuning these embeddings specifically for the dataset could yield even more insightful outcomes.

## Author contributions

AB: Conceptualization, Data curation, Formal analysis, Project administration, Resources, Supervision, Validation, Writing – review & editing. TG: Conceptualization, Data curation, Formal analysis, Investigation, Methodology, Software, Validation, Writing – original draft, Writing – review & editing. YW: Data curation, Writing – review & editing. SS: Data curation, Writing – review & editing. DN: Data curation, Project administration, Resources, Supervision, Validation, Writing – review & editing.

## Footnotes

^1^ lenr-car.org

## Supplementary material

SUPPLEMENTARY FIGURE 1 Unsupervised based document clustering based on research topics.

SUPPLEMENTARY FIGURE 2 Yearly distribution graph of published papers.
