# Supplementary figures and images for: Exploring artificial intelligence techniques to research low energy nuclear reactions
Source: Front Artif Intell. 2024 Aug 23;7:1401782. doi: 10.3389/frai.2024.1401782 (PMC11377257; doi:10.3389/frai.2024.1401782)

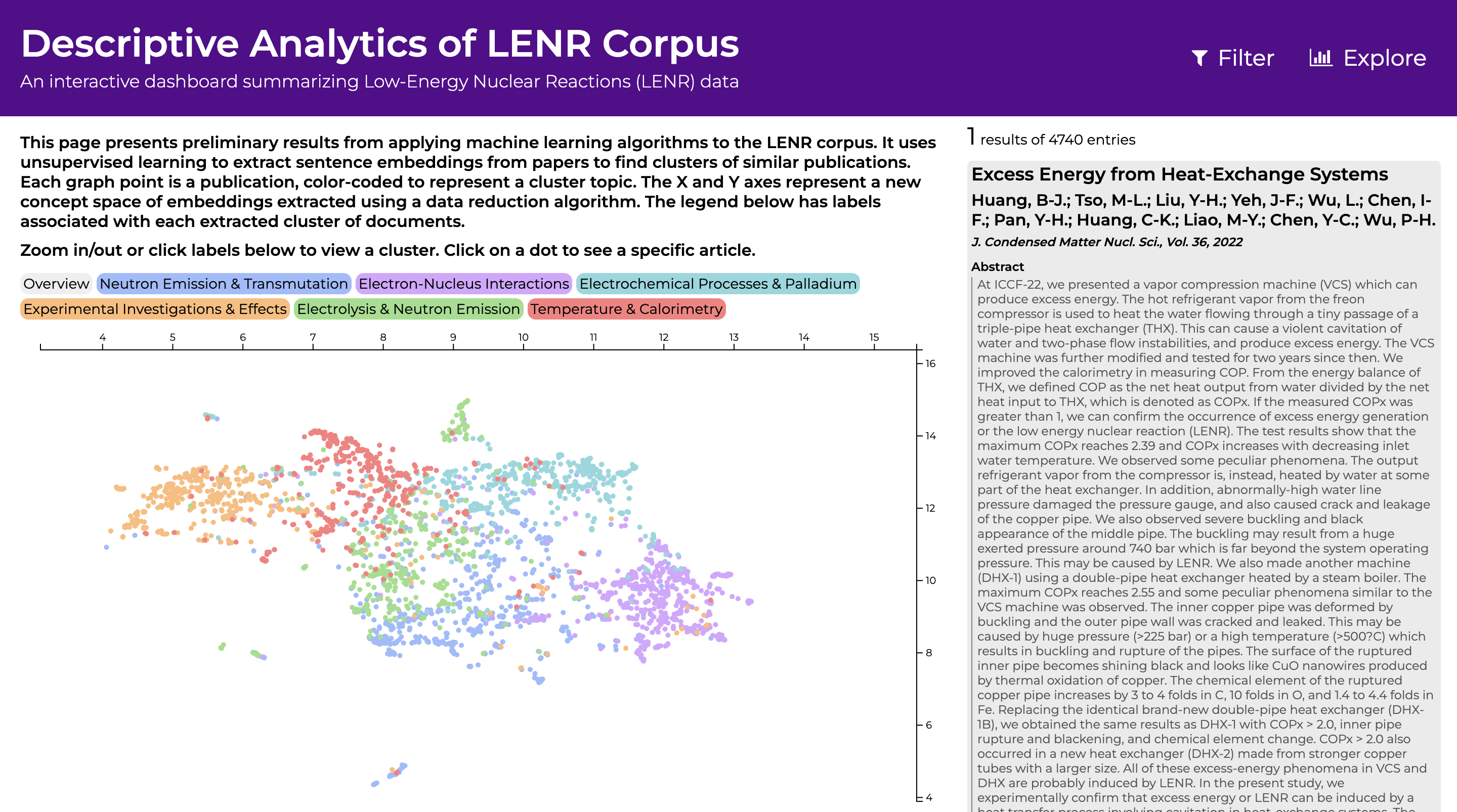

Supplement: SUPPLEMENTARY FIGURE 1 — Unsupervised based document clustering based on research topics. [file Image_1.png]

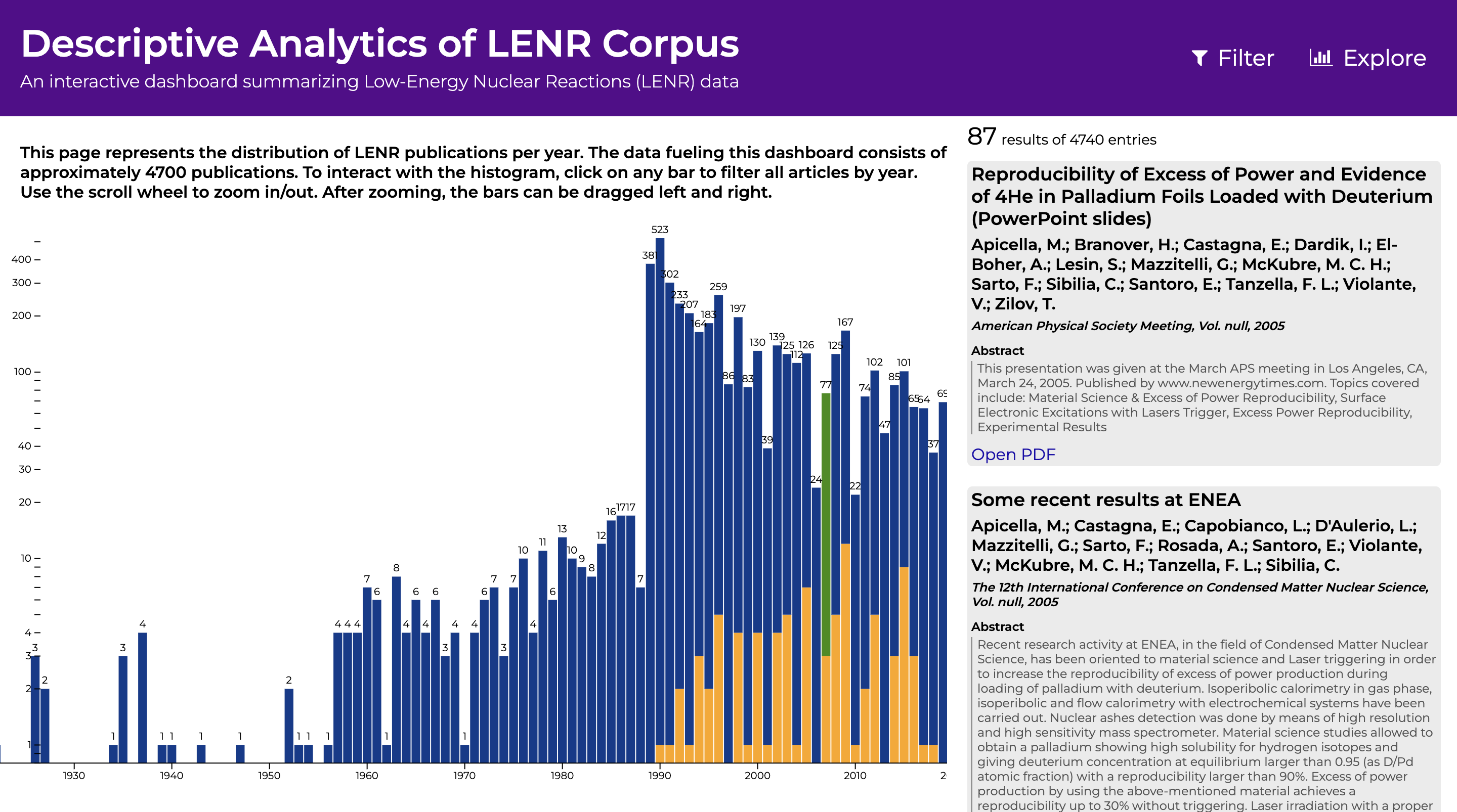

Supplement: SUPPLEMENTARY FIGURE 2 — Yearly distribution graph of published papers. [file Image_2.png]
